# Identification of circular RNAs in porcine sperm and evaluation of their relation to sperm motility

**DOI:** 10.1038/s41598-020-64711-z

**Published:** 2020-05-14

**Authors:** Marta Gòdia, Anna Castelló, Martina Rocco, Betlem Cabrera, Joan Enric Rodríguez-Gil, Sam Balasch, Craig Lewis, Armand Sánchez, Alex Clop

**Affiliations:** 1 Animal Genomics Group, Centre for Research in Agricultural Genomics (CRAG) CSIC-IRTA-UAB-UB, Campus UAB, Cerdanyola del Vallès, Barcelona, Catalonia Spain; 2 Unit of Animal Science, Department of Animal and Food Science, Autonomous University of Barcelona, Cerdanyola del Vallès, Barcelona, Catalonia Spain; 3 Unit of Animal Reproduction, Department of Animal Medicine and Surgery, Autonomous University of Barcelona, Cerdanyola del Vallès, Barcelona, Catalonia Spain; 4 Grup Gepork S.A., Barcelona, Catalonia Spain; 5 PIC Europe, Sant Cugat del Vallés, Catalonia Spain; 6 Consejo Superior de Investigaciones Científicas (CSIC), Barcelona, Catalonia Spain

**Keywords:** Gene expression profiling, Gene expression, Animal breeding

## Abstract

Circular RNAs (circRNAs) are emerging as a novel class of noncoding RNAs which potential role as gene regulators is quickly gaining interest. circRNAs have been studied in different tissues and cell types across several animal species. However, a thorough characterization of the circRNAome in ejaculated sperm remains unexplored. In this study, we profiled the sperm circRNA catalogue using 40 porcine ejaculates. A complex population of 1,598 circRNAs was shared in at least 30 of the 40 samples. Generally speaking, the predicted circRNAs presented low abundances and were tissue-specific. Around 80% of the circRNAs identified in the boar sperm were reported as novel. Results from abundance correlation between circRNAs and miRNAs together with the prediction of microRNA (miRNA) target sites in circRNAs suggested that circRNAs may act as miRNA sponges. Moreover, we found significant correlations between the abundance of 148 exonic circRNAs and sperm motility parameters. Two of these correlations, involving ssc_circ_1458 and ssc_circ_1321, were confirmed by RT-qPCR using 36 additional samples with extreme and opposite sperm motility values. Our study provides a thorough characterization of circRNAs in sperm and suggests that circRNAs hold potential as noninvasive biomarkers for sperm quality and male fertility.

## Introduction

Swine, due to its similarity to humans in its genome sequence, anatomy and physiology, is quickly becoming an important model for biomedical research^1^. In humans, infertility is an increasing problem in contemporary society, affecting one in twenty males^2^, and has become a subject of research in swine^3,4^. While in humans, men attend *In Vitro* Fertilization (IVF) clinics to address infertility issues, the boars at artificial insemination (AI) studs have been previously selected on the basis of their semen quality and consequently, infertility is rare. However, these studs contain subfertile boars and also present phenotypic variation on the semen quality of the animals. Men’s infertility and these phenotypes in the boar studs are expected to share a common molecular basis. Unlike in humans, where fertility data is based on few records per person, the swine industry is currently recording semen quality and fertility phenotypes each time a sire is used for AI. In pig intensive production systems, an AI boar typically inseminates around 1,500 sows during its productive life (S Balasch, pers. comm.). Thus, large datasets of the reproductive ability of these boars is becoming available.

Sperm motility and kinetic parameters provide an objective and reproducible measurement of semen quality that is automatically assessed by the computer-assisted semen analysis (CASA) system. CASA measures among other parameters, the percentage of total motile spermatozoa, the average curvilinear velocity (VCL), the average straight line velocity (VSL) and average path velocity of the sperm cells (VAP) based on trajectories of motile sperm. This approach has been commonly used to assess semen quality in animal breeding prior to AI in cattle^5^, horse^6^ and swine^7^ where significant correlations between sperm motility and fertility have been found. In humans, this technique is also applied to estimate the *in vitro* fertilizing potential of the ejaculates used in assisted reproductive treatments^8^.

Multiple research efforts have demonstrated that sperm quality parameters and fertility outcomes are related to the presence or absence of sperm RNAs. Different studies have provided evidence that the absence or deregulation of certain RNAs is associated to infertility and/or sperm motility in human, mice and cattle^9^. These studies have focused their research not only on messenger RNAs (mRNAs) but also on different classes of non-coding RNAs, including microRNAs (miRNAs) and transfer RNAs (tRNAs)^9,10^.

Circular RNAs (circRNAs) are a novel class of non-coding RNAs with a closed loop structure, mainly formed through pre-mRNA back splicing event^11^. circRNAs are highly stable *in vivo* in comparison to their mRNA linear counterparts, because of their circular structure, which confers protection against exonuclease-mediated degradation^12^. circRNAs mostly have an exonic origin. However, intronic and intergenic circRNAs have also been described^13^. While the first ones are preferentially located in the cytoplasm, the other two are found in the nucleus. Expanding views on their function suggest that circRNAs can directly regulate the abundance of their cognate mRNA and can also act as miRNA sponges^11,12,14^, thus impeding the post-transcriptional inhibitory roles of these miRNAs on their target mRNAs. circRNAs have been identified across tissues in several species including the fruitfly, human, mice and swine, following tissue-specific expression patterns^12^.

During the last few years, there has been a considerable interest in the potential of circRNAs as health biomarkers. Several studies have identified circRNAs which abundances were associated to cancer, aneurysms, hypertension, heart failure, diabetes or arthritis^15^. There are few reports characterizing circRNAs in reproductive organs and cell types including oocyte, embryo, placenta, granulosa cells, immature spermatogenic cells, seminal plasma, testis and more recently, spermatozoa^16–18^. Up to date, a small number of studies have assessed the circRNA predictive potential for reproduction outcomes. Chang and colleagues identified circRNAs associated to embryo quality and suggested their role as potential predictors of live birth^19^. A novel study carried by Chioccarelli *et al*., has characterized the human sperm circRNAome using a circRNA microarray in three normozoospermic donors^18^. In this study, the authors compared, within each sample, two sperm sub-populations defined by their good and bad quality in terms of motility and morphology. This comparison yielded 148 differentially abundant circRNAs between the two semen quality groups^18^.

Our group has recently carried a thorough description of the porcine sperm transcriptome^20^ and the data, in line with previous studies^9^, showed that the majority of the transcripts are highly fragmented and with low abundances. Considering their stability and abundance, circRNAs could hold an important potential as reliable biomarkers for sperm quality and fertility. To shed light into the potential relevance of circRNAs on semen quality, we have carried a *de novo* characterization of the circRNA repertoire in 40 porcine spermatozoa samples and assessed their potential role as miRNA sponges using total and small RNA-seq. Furthermore, we have investigated the correlation between their abundance and sperm motility traits.

## Results

### Phenotypic data

The average percentage of motile spermatozoa was 75.5 with a standard deviation (SD) of 17.4, VCL (mean: 43.7; SD: 10.8), VSL (mean: 26.4; SD: 5.8) and VAP (mean: 32.4; SD: 6.8).

### RNA isolation, RNA-seq and bioinformatics analysis

RNA extraction from 40 mature sperm samples free from somatic cells yielded an average of 2.2 fg per spermatozoa (range between 0.8 and 3.7 fg) (Supplementary Table S1). We obtained an average of 40.7 M total RNA-seq sequencing reads per sample, 98.2% of which passed the quality control filters. After read mapping, the unmapped reads were used for circRNA prediction (Supplementary Table S1). For small RNA-seq, we obtained an average of 7.3 M reads per sample. The vast majority of these reads (99.2%) passed quality controls and 81.5% mapped to the porcine genome (Supplementary Table S1). We identified 95 miRNAs from the list of 306 that are annotated in swine.

### Characterization of the sperm circular RNA repertoire

**Figure 1:**
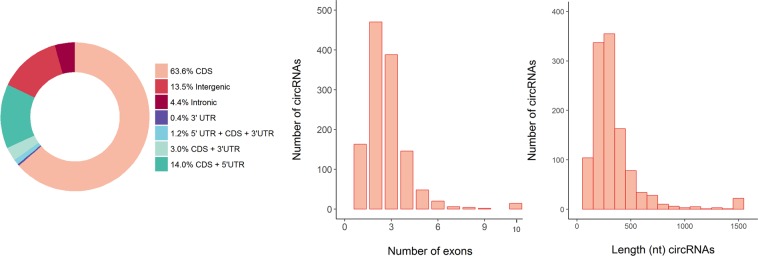
Genomic features of the circRNAs identified in the boar sperm. **(a)** Distribution of the genomic location (CDS, intergenic, intronic, 3′ UTR or 5′UTR) of the 1,598 circRNAs identified in the boar sperm. **(b)** Distribution of the number of exons that form the exonic circRNAs. **(c)** Distribution of the nucleotide length of the exonic circRNAs. CDS: Coding Sequence; UTR: Untranslated Region.

1,598 potential circRNAs were identified as shared in at least 30 of the 40 ejaculates (Supplementary Table S2). The majority of the circRNA species were derived from exonic regions (CDS – Coding Sequence-, 3′ and 5′ UTR –Untranslated Region-) (82.1%), while only 13.5% and 4.4% originated from intergenic and intronic segments, respectively (Fig. 1a). Most circRNAs included less than 4 exons (81.0%), and only 14 circRNAs contained 10 or more exons (Fig. 1b). In addition, the majority of the exonic circRNAs (76.9%) were less than 400 bp long (Fig. 1c). RNA abundances across the different circRNA types were low, with a range between 0.19 and 136.4 Counts Per Million (CPM) and mean and median values of 2.42 and 0.89 CPMs, respectively (Supplementary Table S2). The top 15 most abundant exonic circRNAs encompassed genes related to sperm biology and male fertility including *ATP6V0A2*, *PPA2*, *PAIP2* and *PAXIP1* (Table 1).

**Table 1:** List of the 15 most abundant exonic circRNAs in swine sperm. circRNA genomic coordinates are indicated as chromosome:start_position. .end_position. Mean and SD: standard deviation, are in CPM (Counts Per Million).

circRNA name	circRNA genomic coordinates	Mean	SD	Ensembl ID	Host gene symbol
ssc_circ_1097	4:590305..590427	75.8	51.9	ENSSSCG00000005916	*WDR97*
ssc_circ_1141	5:22008783..22009750	75.0	35.4	ENSSSCG00000000406	*PTGES3*
ssc_circ_1062	4:130322062..130339222	62.2	33.1	ENSSSCG00000006939	*ZNHIT6*
ssc_circ_0537	14:29281863..29290551	59.9	34.8	ENSSSCG00000009766	*ATP6V0A2*
ssc_circ_0777	18:14243637..14259662	47.3	23.9	ENSSSCG00000016529	*AGBL3*
ssc_circ_0860	2:3096936..3098647	45.1	35.6	ENSSSCG00000037451	*PPFIA1*
ssc_circ_0805	18:54260018..54270584	40.0	52.3	ENSSSCG00000035581	*SUGCT*
ssc_circ_0132	1:641933..653683	33.2	19.1	ENSSSCG00000004008	*WDR27*
ssc_circ_1575	AEMK02000682.1:1719140..1720285	28.4	24.6	ENSSSCG00000005753	*CAMSAP1*
ssc_circ_1413	8:116275909..116294261	26.9	15.5	ENSSSCG00000022788	*PPA2*
ssc_circ_0895	2:64840342..64840811	25.8	17.5	ENSSSCG00000013776	*DDX39A*
ssc_circ_1101	4:6367950..6392035	22.6	28.8	ENSSSCG00000005941	*KHDRBS3*
ssc_circ_0839	2:141254413..141254577	21.8	20.9	ENSSSCG00000026606	*PAIP2*
ssc_circ_0795	18:3101570..3102902	21.7	9.0	ENSSSCG00000025221	*PAXIP1*
ssc_circ_0021	1:106150520..106171073	20.26	11.02	ENSSSCG00000004538	*WDR7*

Only a small fraction of genes produced more than one circRNA. These were considered circRNA hotspots. The circRNAs from hotspot genes are often produced from different back-splicing events of one exon with several others^21^. We detected 12 genes with 5 or more circRNA isoforms (Table 2). Again, four of these genes, namely *TESK2*, *SPATA19*, *PTK2* and *SLC5A10* have been directly related to sperm function and fertility.

**Table 2:** circRNA hotspot genes in swine sperm. 12 genes harbored 5 or more exonic circRNAs.

Gene symbol	Ensembl ID	Number of circRNAs
*DENND1B*	ENSSSCG00000010900	8
*DENND1A*	ENSSSCG00000005585	7
*TESK2*	ENSSSCG00000003917	6
*UIMC1*	ENSSSCG00000022508	6
*ARMC9*	ENSSSCG00000023994	5
*CAMSAP1*	ENSSSCG00000005753	5
*KDM5B*	ENSSSCG00000010928	5
*PTK2*	ENSSSCG00000038397	5
*RPS6KC1*	ENSSSCG00000015586	5
*SPATA19*	ENSSSCG00000025612	5
*SLC5A10*	ENSSSCG00000018049	5
*WNK1*	ENSSSCG00000000753	5

### Sperm circRNAs might be involved in epigenetic regulation and spermatogenesis

We analyzed the potential roles of the boar sperm circRNAs under the assumption that their function is related to their known mRNA counterpart. Gene Ontology (GO) analysis of the circRNA host genes revealed an enrichment for epigenetic functions including histone modification (q-val: 5.52 ×10^−6^), histone H3-K36 methylation (q-val: 8.65 ×10^−3^) and chromatin organization (q-val: 2.16 ×10^−8^) (Supplementary Table S3). Other significantly enriched ontologies included among others, spermatogenesis (q-val: 5.81 ×10^−4^), cilium assembly (q-val: 4.15 ×10^−3^) and developmental process (q-val: 1.33 ×10^−2^) (Supplementary Table S3).

### The boar sperm has a highly specific circRNAome

We compared our circRNA catalogue with equivalent available datasets from other studies in human (including different brain sections and cell lines)^22^, mice (several brain segments, cell types and embryonic stem cells)^22^ and swine (lung, skeletal muscle, fat, heart, liver, spleen, kidney, ovary, testis and 5 brain sections)^21,23^. Twenty-four % and 11.3% of the boar sperm circRNAs had potential orthologs in human and mouse, respectively (Table 3). On the other hand, 20.3% of the porcine sperm circRNAs were also present in other porcine tissues^21,23^ (Table 3). The porcine tissues showing higher overlap with sperm were testes (11.6%) and brain cortex (11.3%) (Table 3). Comparing this in the opposite direction, 4.9% (6,636 circRNAs) of the circRNAs already annotated in any pig tissue were also present in our pig sperm list. This value was 12 times lower (0.4%) when evaluating the much bigger (90,067 circRNAs) human circRNA catalog (Table 3).

**Table 3:** Concordance between the circRNAs catalogue of the boar sperm and the circRNA list in other species and pig tissues. Number of circRNAs after Sscrofa11.1 liftover from human, mice and swine (detailed by tissue). Column ‘Pig sperm’ shows the proportion of swine sperm circRNAs that were identified in the given species and tissues. Column ‘Other species/tissues’ of the total circRNAs lists the proportion of circRNAs from that tissue or species that found a homolog in the boar sperm.

Species/Tissues	Number of circRNAs	Pig sperm	Other species/tissues
Human	90,067	24.0%	0.4%
Mice	15,498	11.3%	1.2%
Swine	6,663	20.3%	4.9%
Basal ganglia	456	2.6%	9.2%
Brain stem	820	5.3%	10.2%
Cerebellum	1,061	5.6%	8.4%
Cortex	2,163	11.3%	8.4%
Hippocampus	549	3.2%	9.3%
Fat	494	2.4%	7.7%
Heart	539	2.2%	6.5%
Kidney	469	1.9%	6.4%
Liver	353	2.1%	9.3%
Lung	683	2.7%	6.3%
Muscle	532	2.6%	7.9%
Ovarium	652	3.8%	9.4%
Spleen	241	1.4%	9.1%
Testes	2,685	11.6%	6.9%

### Sperm circRNAs do not follow an age-dependent pattern

We assessed whether sperm circRNAs depicted an age-accumulating profile as it has been previously observed in rat testes^24^, and rat and mouse brains^24,25^. All the boars from our dataset were sexually mature with ages ranging between 9 and 54 months of age. Sexual maturity in boars is a process that starts at the age of 8 months and finalizes when animals reach 2 years. Thus, we divided the samples in those coming from boars approaching sexual maturity with ages below 2 years old and those produced by mature pigs with ages above 2 years old. There was no significant difference in the number of circRNAs identified (*P*-value: 0.68, Wilcoxon rank sum test) nor in their RNA abundance (*P*-value: 0.948) between the two groups. We repeated the analysis considering only extreme ages: young (N = 4; between 8.6 and 9.2 month old) and mature (N = 4; 29.9 to 54.6 month old). Again, there was no difference in the number of circRNAs identified (*P*-value: 0.89) or in their abundance (*P*-value: 0.2).

### circRNA-miRNA interaction network

**Figure 2:**
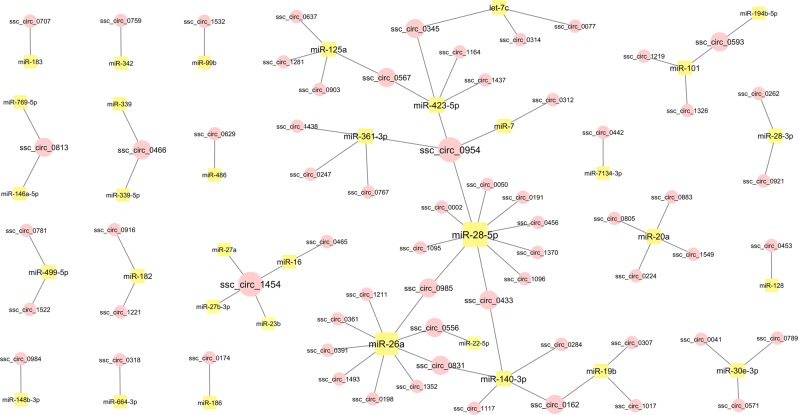
CircRNA-miRNA interaction network. circRNA:miRNA relationships predicted by both RNA co-abundance and identification of miRNA target sites in the circRNAs sequences. Circular and square nodes represent circRNAs and miRNAs, respectively. The node and letter sizes indicate the number of significant correlations involving the node.

To evaluate the potential role of exonic circRNAs as miRNA sponges, we built a circRNA:miRNA co-expression network with the RNA abundances of the 1,261 exonic circRNAs (from total RNA-seq libraries) and the 95 miRNAs (from short RNA-seq libraries). The analysis identified 1,882 significant correlations that included 95 miRNAs and 458 exonic circRNAs. In parallel, we also performed an *in silico* prediction of miRNA target sites involving the miRNAs and exonic circRNAs identified in our data. We assessed miRNA target sites in the exonic circRNA sequences based on sequence complementarity using the tool miRanda^26^. The analysis yielded 4,987 potential targets involving all the 95 miRNAs and 1,103 circRNAs. To reduce the proportion of false-positives, only the 81 interactions (from 34 miRNAs and 65 circRNAs, each from a different gene) that were identified in both approaches were used for network visualization (Fig. 2). Fifty-three of these 65 circRNAs were predicted to interact with only one miRNA (Fig. 2). Twelve circRNA were predicted to interact with two or more miRNAs. The circRNAs displaying the largest number of predicted interactions with miRNAs were ssc_circ_0954 and ssc_circ_1454, which connected with four miRNAs each (Fig. 2). On the other hand, miR-28-5p and miR-26a were predicted to be regulated by 10 and 9 different circRNAs, respectively. Similarly, miR140-3p and miR-423-5p were potentially targeted by five different circRNAs each (Fig. 2).

### Correlation of circRNAs with sperm motility and circRNA validation

A total of 148 exonic circRNAs from 142 genes showed significant correlations between their abundance and sperm motility traits (Supplementary Table S4). More in detail, 24, 83, 24 and 41 circRNAs correlated with the percentage of total motile spermatozoa, VCL, VAP and VSL, respectively (Supplementary Table S4).

To confirm the existence of these circRNAs and their phenotypic correlations we undertook a Reverse Transcription quantitative PCR (RT-qPCR) and a Sanger Sequencing approach. First, we randomly selected two circRNAs with different RNA abundance levels to test whether we could confirm the bioinformatically predicted circRNAs. The chosen circRNAs were: ssc_circ_1141 (from *PTGES3*) with 75.0 CPM, and ssc_circ_0670 (from *BAZ2B*) with 10.6 CPM, both also detected in human^22^ (hsa_circ_0008137 and hsa_circ_0002463, respectively) and in swine testes^23^. The PCR amplification of the two circRNAs resulted in a single electrophoretic band of the expected size (Supplementary Fig. S1a) and Sanger Sequencing confirmed the back-splice junction (BSJ) (Supplementary Fig. S2a,b).

**Figure 3:**
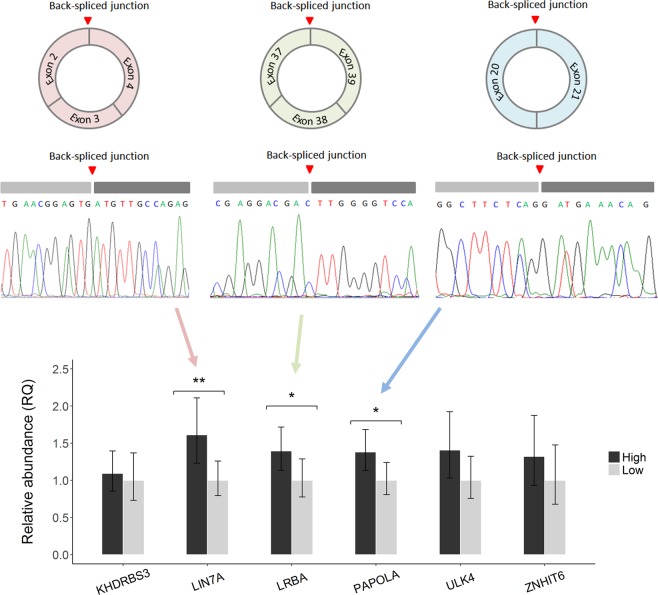
Validation of the circRNAs which RNA-seq based abundance correlated with sperm motility. **(a)** Sanger sequencing validation of the circRNA black splice junction for ssc_circ_1132 from *LIN7A*, ssc_circ_1458 from *LRBA* and ssc_circ_1321 from *PAPOLA*. **(b)** Relative abundance of six circRNAs in samples with extreme and divergent motility values (18 samples displaying high motility and 18 samples with low motility) obtained by RT-qPCR. The RNA-seq based association between ssc_circ_1458 and ssc_circ_1321 abundance and sperm motility was validated by RT-qPCR in the 36 samples.

Then, we used the same approach to confirm the existence of 8 circRNAs selected for their significant correlation with at least one motility trait (Supplementary Table S4). These circRNAs were further selected based on the fact that: (i) they were present at high abundances, or (ii) they showed significant correlation with at least 1 trait, or (iii) they had been previously identified in pig^23^, human^22^ or mice^22^. PCR amplification of 6 of the 8 circRNAs resulted in a band of the expected size (Supplementary Fig. S1b) and the Sanger Sequencing validated the expected BSJ (Fig. 3a; Supplementary Fig. 2c-e). ssc_circ_0839 from *PAIP2* did not amplify (data not shown) and ssc_circ_0118 from *PDE10A* (also identified in pig testes and in human as hsa_circ_0078638) displayed 2 amplification bands (Supplementary Fig. S1b). These two circRNAs were discarded for further analysis. Thus, we confirmed the existence of these six circRNAs.

The RT-qPCR levels of the 6 circRNAs were measured in 36 animals presenting extreme and opposite values of sperm motility (N = 18 for each phenotypic distribution tail) from a dataset of 300 boar ejaculates with phenotype records. None of the 36 samples was included in the RNA-seq study. The two sample groups displayed significant phenotypic differences for all the traits: percentage of total motile spermatozoa (*P*-value: 2.65 ×10^−9^, Wilcoxon rank sum test), VCL (*P*-value: 2.20 ×10^−10^), VSL (*P*-value: 3.22 ×10^−7^) and VAP (*P*-value: 3.22 ×10^−7^). The RT-qPCR assays presented efficiencies between 99.6% and 105.2%.

Two of the six circRNAs showed significant differences between the 2 sperm motility groups (Fig. 3b). These 2 circRNAs were ssc_circ_1458 from *LRBA* (*P*-value: 0.049) and ssc_circ_1321 from *PAPOLA* (*P*-value: 0.035). A third circRNA, ssc_circ_1132, from *LIN7A*, showed significant differences between the two motility groups (*P*-value: 0.008) (Fig. 3b) but in the opposite direction than expected according to the RNA-seq data and was consequently considered as not validated due to inconclusive results. The other 3 circRNAs, ssc_circ_1101 from *KHDRBS3*, ssc_circ_0437 from *ULK4* and ssc_circ_1061 from *ZNHIT6* did not present significant differences between the 2 groups (Fig. 3b).

## Discussion

This study provides the first RNA-seq based circRNA repertoire of mature spermatozoa in a mammalian species. We have also assessed the sperm circRNA role as miRNA sponges and evaluated their associations with sperm motility as a parameter of semen quality. Our results provide further evidence and expand the relevance of spermatozoa RNAs in sperm biology and quality, as recently described in other studies^18^. circRNAs have been reported as promising potential biomarkers for human health including cancer, diabetes, cardiovascular diseases or the pathogenesis of pre-eclampsia^15^. Here, we provide novel data supporting the involvement of circRNAs on the male’s reproductive function as reflected by their association with several sperm motility parameters.

We have identified nearly 1,600 boar sperm circRNAs that are robustly present in most samples (at least 30). The sperm circRNA repertoire showed similar circular genomic characteristics including the proportion of genomic overlap with protein coding and intergenic regions, the number of exons and the circRNA length (Fig. 1), when compared to data previously reported in other tissues from swine^23^, human^17^ or rat^24^. Nonetheless, the list of boar sperm circRNAs showed a modest overlap with these other datasets (Table 3). Not surprisingly, the largest overlap was with porcine testes (11.6%), probably, due to the fact that the male gonads are mainly composed of cells from the spermatogenic lineage including spermatozoa. The reduced concordance between swine sperm and other tissues and species (Supplementary Table S5) may be partly influenced by technical differences involving the processing of samples and the data analysis between the studies. Moreover, opposite scenarios were identified in swine and human. In swine, there was high sperm-specificity, with nearly 80% of the circRNAs being present only in sperm (Table 3). In contrast, human sperm gave a positive signal for the majority (72%) of the 13,617 circRNAs from circBase^22^ that were queried using microarray technology^18^. A biological explanation for this difference between swine and human cannot be rule out but it is unlikely. The mRNA and miRNA components and payload of the human and porcine sperm have been previously interrogated and are highly ressemblant^20^. For this reason, we hypothesize that the differences observed are merely technical and related to the processing of the samples and the data.

We investigated the functional relevance of sperm circRNAs under the hypothesis that their function is associated to the host gene. Four genes (*ATP6V0A2*, *PPA2*, *PAIP2* and *PAXIP1*) harboring the 15 most abundant exonic circRNAs (Table 1) have been directly implicated to sperm related traits and male fertility. The vacuolar ATPase *ATP6V0A2* transcripts and proteins are down-regulated in the sperm of infertile men^27^. The pyrophosphatase *PPA2* is located in the mitochondrial membrane and might be involved in the production of ATP, control of molecular processes linked to the launching of sperm capacitation and sperm motility^28^. *PAIP2* is an inhibitor of translation and is linked to sperm maturation and male fertility^29^. Finally, *PAXIP1* is critical for genome stability and chromatin condensation and is associated with developmental arrest of spermatocytes, testicular atrophy and infertility in knockout mice^30^. We also identified 12 hotspot genes producing five or more circRNAs each (Table 2). Some of these hotspot genes were related to sperm function and fertility. *TESK2* is a protein kinase mostly expressed in round spermatids and is predicted to play a role in early stages of spermatogenesis^31^. *PTK2*, is essential for embryo development^32^. *SPATA19* is critical for sperm mitochondrial function in relation to sperm motility and fertilization ability^33^.

The ontology enrichment analysis of the genes harboring circRNAs pointed towards epigenetic related functions, which are essential for chromatin condensation in sperm and for the reprogramming of gene expression upon egg fertilization and during embryo development (Supplementary Table S3). Gene enrichment analysis also signaled towards spermatogenesis and developmental processes, also involving the embryo development related genes, *DHX36, IPMK, RICTOR, CDC73* and *ANGPT1* (Supplementary Table S3). These functions are in line with previous works studying sperm mRNAs^9,20^, thereby providing further basis for the regulatory role that circRNAs might have on their cognate linear mRNAs.

A previous study in rat testes identified a dynamic circRNA age-dependent pattern of expression and suggested a relation between their abundance and function with the male’s sexual maturity and spermatogenesis^24^. For this reason, we sought to investigate whether, like in rat testes, circRNAs also accumulate through age in the porcine sperm. Our data suggested that there is no association between the mature sperm circRNAs and the boar’s age. This could be explained by different scenarios. First, while the study on rat testes compared animals with broad age differences (between 2 and 104 weeks)^24^, all our samples were from adult pigs with less dissimilar ages (between 9 and 54 months) taking into account that swine has a longer life cycle. Second, the study in rats assessed the whole testis payload, which includes cells from the spermatogenic lineage and also, leukocytes, endothelial, Sertoli and Leydig cells. Our study in pigs exclusively queried mature-selected ejaculated spermatozoa. Cells with high proliferation rates^34^ such as spermatogonial stem cells (SSC) have been suggested to accumulate less circRNAs due to passive thinning out during their continuous self-renewal and proliferation until they become spermatozoa. Spermatozoa originates from SCC through a highly constant and orchestrated process and upon maturation, they enter a silent state with no apparent transcriptional activity. This would imply that in spermatozoa, circRNAs do not accumulate through age. Interestingly, our results indicate that circRNAs in mature sperm are stable and thus, provide further support for the potential of circRNAs as noninvasive biomarkers for male reproductive conditions.

To shed light into the functional relevance of circRNAs as miRNA sponges we built an interaction network (Fig. 2). We combined RNA-seq benchwork data of circRNA and miRNA abundances and *in silico* prediction of miRNA target sites to increase the reliability of the circRNA-miRNA relationship predictions. This network of 81 interactions contained some circRNA genes and miRNAs previously related to semen quality and male fertility. Remarkably, 2 circRNAs, ssc_circ_0954 and ssc_circ_1454 were linked to 4 different miRNAs each. The first, ssc_circ_0954 arises from *DCDC2C*, a gene identified in the human sperm flagellum end-piece with a suggested role on microtubule dynamics by acting as a depolymerization/polymerization balancing system^35^. This circRNA was predicted to interact, among others, with miR-361-3p which was found to be dysregulated in subfertile men^36^, miR-423-5p, which was altered in oligozoospermic men^37^ and miR-28-5p, a miRNA that was dysregulated in normozoospermic infertile individuals^38^. The other cirRNA, ssc_circ_1454, is transcribed from *MTHFD2L*, a mitochondrial isozyme from the folate cycle metabolic pathway, a vitamin that has been also related to semen quality and fertility in men^39^. One of the miRNAs targeted by ssc_circ_1454 was miR-16, which was dysregulated in subfertile men^36^. The network also showed 10 circRNAs that may be regulating miR-28, a miRNA (miR-28-5p) that was dysregulated in normozoospermic infertile individuals^38^. The potential miR-28 regulators included ssc_circ_1370, arisen from *FAM92A*, whose protein may play a role in ciliogenesis^40^, and ssc_circ_0002 from *WDR7*, which is associated to sperm quality in cattle^41^. Likewise, 9 circRNAs were predicted to regulate miR-26a, which has been in turn, linked to VCL, VSL and VAP motility parameters in a previous study in swine^42^. miR-26a was also targeted by ssc_circ_0361, a circRNA from *ACTL6A*, which is a gene with a crucial function for embryo development^43^ and ssc_circ_1352 from *CAGE1*, an acrosomal protein with proposed roles in fertility^44^. Additional interesting interactions included ssc_circ_0345 from the hotspot gene *SLC5A10* (sodium-dependent mannose and fructose transporter) (Table 2), which regulated miR-423-5p, a miRNA that was upregulated in oligozoospermic semen^37^ and let-7c, which was altered in patients with severe asthenozoozpermia^45^. Altogether, the network involved host genes and miRNAs that are connected to male fertility thereby suggesting that circRNAs may play a role in the male’s reproductive ability.

The potential role of circRNAs in male reproduction is further substantiated by the associations detected between sperm motility and the abundance of some sperm circRNAs in our study and the work detailed by Chioccarelli and co-authors^18^. At least 20 of the circRNA host genes implicated in the phenotypic correlations have been previously linked to sperm biology or male fertility (Supplementary Table S4). For example, ssc_circ_0823, which correlated with VCL (*P*-value = 0.009), is a circRNA hosted by *CAMK4*, a gene that has been implicated in sperm motility in humans^46^. ssc_circ_0780, correlated with the percentage of motile cells (*P*-value = 0.041), is hosted by *LRGUK*, a gene that is required for sperm assembly including the growth of the axonome, a key structure for the flagellar beating in sperm^47^. We successfully tested by RT-qPCR 6 of the 148 exonic circRNAs that correlated to sperm motility. We validated their correlation with motility traits for 2 of these 6 circRNAs, ssc_circ_1458 from *LRBA* and ssc_circ_1321 from *PAPOLA*. Both were significantly down-regulated in the ejaculates with low sperm motility (Fig. 3b). *LRBA* is a gene involved in coupling signal transduction and vesicle trafficking but no link with sperm function or fertility has been found thus far. ssc_circ_1321 from *PAPOLA*, has a human ortholog circRNA (hsa_circ_0033126) and the host gene is implicated in RNA and ATP binding. Thus, none of the two host genes have been previously related to sperm function or fertility. However, the association is confirmed by RT-qPCR and we cannot exclude unidentified relevant functions on sperm motility. Two other circRNAs were also of high interest and tested as they were within the top 15 most abundant (Table 1) and correlated with sperm motility (Supplementary Table S4). They were ssc_circ_0839 from *PAIP2*, with crucial roles in spermatogenesis^29^ and ssc_circ_1101 from *KHDRBS3*, a gene that is highly abundant in mice testes^48^. ssc_circ_0893 did not amplify when subjected to RT-qPCR and ssc_circ_1101 did not present significant differences in the RT-qPCR levels between the motility groups (Fig. 3b).

Interestingly, some circRNAs popped up as relevant in more than one of the analyses carried in this study. For example, ssc_circ_1532, from the *SPATA19* hotspot gene (Table 2), which is related to sperm motility and fertility^33^, was suggested to regulate miR-99a according to the network analysis (Fig. 2). miR-99b was found to be dysregulated in the low motility sperm fraction in bull^49^ and in subfertile men^36^. Another circRNA, ssc_circ_1219 from *OSBPL9*, a gene involved in male reproduction^50^, displayed abundance correlation with VCL (*P*-value: 0.04) (Supplementary Table S4) and was identified as a potential target of miR-101 (Fig. 2), a miRNA (miR-101-3p) that was altered in asthenozoospermia men^45^.

Remarkably, 4 (*DENND1B*, *PTK2*, *SLC5A10* and *CAMSAP1*) of the 12 hotspot genes hosted a circRNA with significant abundance correlation with sperm motility. Thus, one third of the hotspot genes included circRNAs correlated with sperm motility whilst only 15.0% (148) of the 984 genes hosting the 1,598 circRNAs were correlated with motility (Supplementary Table S4). Noteworthy, the 12 circRNA hotspot genes were not hotspots in the other porcine tissues analyzed^21,23^ (data not shown). Altogether, this indicates that circRNA hotspot genes may have relevant tissue-specific functions.

In conclusion, our study is the first to characterize the circRNAs present in porcine spermatozoa. We have provided a comprehensive view of the boar sperm circRNAome, which is highly sperm-specific and involves genes related to sperm biology and development and identified potential circRNAs acting as miRNA sponges. Moreover, we have detected and validated correlations between the abundance of some circRNAs and sperm motility parameters. The results described in this study may be of interest for animal breeding and for human health. On the one side, our data identified genomic regions of relevance for sperm quality that could harbor SNP markers for the early prediction of the piglets that will enter the AI studs. On the other hand, this study may spur novel research on the potential of circRNAs as noninvasive biomarkers for male fertility in human medicine.

## Methods

### Sperm collection and phenotyping

An integrated figure of the study design can be found in Supplementary Fig. S3. Forty ejaculates, each from a different Pietrain boar, were obtained from GEPORK and from Semen Cardona (Catalonia) for this study. The ejaculates had been collected using the hand glove method by trained professionals under the routine sperm sampling using their relevant procedures and guidelines. No animal experiment has been performed in the scope of this research. Fresh sperm motility traits were assessed with the CASA system (Integrated Sperm Analysis System V1.0; Proiser, Valencia, Spain). In this study we analyzed sperm motility parameters, including the total percentage of motile cells (VAP > 10 µm/s), VCL (µm/s), VSL (µm/s) and VAP (µm/s). Phenotypes were corrected for the fixed variables: farm (1, 2, 3), age (1, 2, 3) and season and year (Autumn 2014, 2015 and 2016; Winter 2015, 2016 and 2017; Spring 2015 and 2016; Summer 2015) using the R function “lm”^51^.

The 40 ejaculates were used for RNA-seq and 34 of them for short RNA-seq. The ages of these boars ranged between 9 and 54 months, with average and median of 15 and 11 months old, respectively. Twenty-four boars were less than 1 year old, 12 pigs were between 1 and 2 years old, 3 pigs between 2 and 3 years old (Supplementary Table S6).

### RNA isolation and library preparation

Ejaculates were purified to remove somatic cells and immature sperm cells and purified sperm was stored at −96 °C with Trizol^®^ as described by Gòdia *et al*.^52^. Briefly, the ejaculates were suspended over a 3 ml cushion of the commercial solutions of BoviPure and BoviDiluteTM (Nidacon; Mölndal, Sweden), which includes colloidal silica particles coated with silane in an isotonic salt solution in a 15 ml RNAse-free tube. Then, the samples were subjected to centrifugation at 300 × g for 20 min at 20 °C. After centrifugation, all the upper phases were removed and the cell pellets were transferred to a new RNase-free 15 mL tube, washed with 10 mL of RNase-free PBS and centrifuged at 1,500 × g for 10 min at 20 °C. The supernatants were then removed and the pellets optically inspected using a microscope to confirm the removal of somatic cells. RNA was extracted from purified sperm cells, treated with TURBO DNA-free™ Kit (Invitrogen) and quantified using Qubit^TM^ RNA HS Assay kit (Invitrogen). We assessed RNA integrity with the 2100 Bioanalyzer and yield using the Agilent RNA 6000 Pico kit (Agilent Technologies). We then performed RT-qPCR assays for *PRM1* and *PTPRC* mRNAs as well as for intergenic/genomic DNA to verify proper sperm purification.

Forty sperm RNA samples were subjected to total RNA-seq. Thirty-four of these samples were also used for small RNA-seq. For total RNA-seq libraries, ribosomal RNA (rRNA) was depleted with the Ribo-Zero Gold rRNA Removal Kit (Illumina) and libraries were constructed with the SMARTer Universal Low Input RNA library Prep kit v2 (Clontech). Resulting libraries were sequenced in a HiSeq2000/2500 system (Illumina) to generate 75 bp long paired-end reads. For small non coding RNA-seq libraries, extracted RNA without rRNA depletion was directly subjected to library preparation with the NEBNext Small RNA Library Prep Set kit (New England Biolabs) and sequenced on a HiSeq2000 (Illumina) to generate 50 bp single-end reads.

### RNA-seq and bioinformatics analysis

For the total RNA fraction, raw reads were filtered by removing adaptor sequences and low-quality reads with Trimmomatic v.0.36^53^. The identification of circRNAs was carried on these reads with the find_circ pipeline^14^ with stricter filter stringency. To reduce the false positive rate in the discovery of circRNAs, we selected circular splice transcripts with at least two unique supporting reads in the anchor segment and with Phred quality scores of 35 or more. Moreover, only circRNAs predicted in at least 30 samples were kept. The RNA abundance of the predicted circRNAs were normalized as the number of BSJ spanning reads per million raw reads (CPM)^21^. The functional regions of circRNAs were identified based on their co-location with genomic features (e.g. exon, 3′UTR, 5′UTR, etc) from the Ensembl database (release 91) with BEDtools^54^. Our catalogue of boar sperm circRNAs was contrasted with other publically available porcine circRNA databases including heart, liver, spleen, lung, kidney, ovarium, testis, skeletal muscle, fat and fetal brains^21,23^. We also queried several human tissues (including several cell lines, brain sections placenta, muscle, fat, umbilical cord, atrium, decidua and plasma) and murine (cell lines and brain sections) available at the circBase database^22^. Genomic coordinates from the human and mouse circRNAs were liftover to Sscrofa11.1 using the UCSC liftover tool^55^.

For the small RNA-seq analysis, trimming of adaptors and low quality bases was performed with Cutadapt v1.0^56^. The mapping of sncRNAs was performed with the sRNAtoolbox v.6.17^57^ with default settings and providing miRBase^58^ release 21 as library dataset. Multi-adjusted read counts were then normalized by sequencing depth as CPM. We only considered the miRNAs that were detected >1 CPM in all the samples.

### circRNA-miRNA network visualization

To identify circRNA:miRNA interactions, we carried a Partial Correlation with Information Theory (PCIT) analysis^59^ using the RNA abundance levels of the of exonic circRNAs and miRNAs after stabilization with log2 transformation. Only negative RNA abundance correlations between circRNAs and miRNAs were kept. We further assessed circRNAs-miRNAs interactions using miRanda v.3.3a^26^ to predict miRNA target sites in the circRNAs sequences. The potential interactions identified with both approaches were kept and visualized with Cytoscape v.3.7.0^60^.

### Gene Ontology analysis and correlation with sperm motility parameters

GO analysis was carried with PANTHER v.13.1^61^ with the overrepresentation test and *P*-values corrected with FDR. Annotation Data Set was “GO biological process complete”. Pearson correlation was used to determine associations between circRNA abundance levels and sperm motility parameters. *P*-values < 0.05 were considered statistically significant.

### Validation of circRNAs and reverse transcription quantitative PCR (RT-qPCR)

circRNAs were validated by Sanger Sequencing and quantified by RT-qPCR using divergent primers. Primers were designed using the Primer Express software (Applied Biosystems). Primer sequences are shown in Supplementary Table S7. For cDNA synthesis, 5 µl of RNA were reverse transcribed using the High Capacity cDNA Reverse Transcription kit in a final volume of 50 μL (Applied Biosystems) following the manufacturer’s protocol. circRNAs were amplified and visualized in 3% high resolution agarose gel electrophoresis and confirmed by Sanger Sequencing.

The abundance level of 6 circRNAs, correlated to sperm motility parameters in the RNA-seq study, was analyzed by RT-qPCR in 36 samples, none of them included in the RNA-seq. These 36 samples belong to two groups with extreme and divergent values for sperm motility from a bank of 300 ejaculates with phenotypic records. Isolated RNA could not be treated with RNAse R due to extremely low RNA yield (see Supplementary Table S1), sample availability and treatment optimization. Nevertheless, one of the circRNAs was designed in the BSJ (Supplementary Table S7) and amplified successfully; which is suggestive of the true circular structure of the RNA products identified by RNA-seq and validated by RT-PCR. Moreover, some reports suggest RNase R can introduce technical noise by degrading some circRNAs while some linear transcripts are resistant to digestion in their 3′ ends^62^.

Quantitative PCR reactions were performed in triplicate following the manufacturer’s instructions. The final reaction (15 μL) included 7.5 μL SYBR Select Master Mix (Life Technologies -Thermo Fisher Scientific), 300 nM of each primer and 3.75 μL of cDNA 1:4 diluted on a QuantStudio 12K Flex Real-Time PCR System (Applied Biosystems). To evaluate the efficiency of the RT-qPCR assays, standard curves with 6 serial dilutions from a pool of sperm cDNA were generated. Thermal profile was set as follows: 50 °C for 2 min, 95 °C for 10 min and 40 cycles at 95 °C for 15 sec and 60 °C for 60 sec. Moreover, a melting profile (95 °C for 15 sec, 60 °C for 15 sec and a gradual increment of temperature with a ramp rate of 1% up to 95 °C) was programmed at the end of the RT-qPCR to assess the specificity of the reactions. The genes *ISYNA2* and *GRP137* were selected as endogenous controls following the stability values after a GeNorm pilot experiment. Their stability was determined considering a GeNorm M value <0.5. Relative expression values were calculated using the ThermoFisher Cloud software (Applied Biosystems) applying the 2^-ΔΔCt^ method. The same software was used to compare the biological groups. Significance was set at a *P*-value < 0.05.

## Supplementary information

Supplementary Information.

Supplementary Tables.

## Data Availability

The datasets generated and/or analysed during the current study are available at NCBI’s BioProject PRJNA520978. Sample phenotypes can be provided upon reasonable request.
