# Supplementary material for: Identification of circular RNAs in porcine sperm and evaluation of their relation to sperm motility
Source: Sci Rep. 2020 May 14;10:7985. doi: 10.1038/s41598-020-64711-z (PMC7224279; doi:10.1038/s41598-020-64711-z)

Title:

Identification of circular RNAs in porcine sperm and evaluation of their relation to sperm motility.

Authors:

Marta Gòdia

Anna Castelló

Martina Rocco

Betlem Cabrera

Joan Enric Rodríguez-Gil

Sam Balasch

Craig Lewis

Armand Sánchez

Alex Clop

Supplementary Information

Supplementary Table S1. Average and Standard Deviation (SD) of the RNA yields, and sequencing and mapping statistics for total and small RNA-seq.

Supplementary Table S2. List of the 1,598 circRNAs identified in sperm with their genomic coordinates, mean abundance (in CPM) and Standard Deviation (SD) in the 40 samples, and the host gene of the exonic circRNAs.

Supplementary Table S3. Gene Ontology analysis and FDR value of the circRNA host genes.

Supplementary Table S4. Correlation between circRNA abundance and sperm motility parameters. The table includes information on the genomic coordinates of the circRNAs, *P*-values of the correlation with sperm motility parameters, host gene, whether it was tested for RT-qPCR validation, and the article reference for these host genes that have previously been associated to sperm biology or male fertility. MT: total percentage of motile cells; VCL: curvilinear velocity; VSL: straight line velocity; VAP: velocity of the sperm cells; ns: not significant.

Supplementary Table S5. Concordance on the list of circRNAs present in 15 porcine tissues.

Supplementary Table S6. Age of the 40 boars at the time of ejaculate collection.

Supplementary Table S7. List of primers designed and used for the RT-qPCR to assess the abundance of target circRNAs and reference genes.

Supplementary Figure S1. Figure displaying the validation of the amplified set of circRNAs by agarose-gel electrophoresis. **A.** Amplification of the 2 randomly selected circRNAs. Two different primer sets for ssc_circ_1141 from *PTGES3* were tested (primer pair a in lane 2 and b in lanes 3 and 4) and ssc_circ_0670 from *BAZ2B* (lane 5)*.* **B.** Validation of the circRNAs correlated to sperm motility parameters: ssc_circ_1321 from *PAPOLA* (*ENSSSCG00000002505),* ssc_circ_1458 from *LRBA,* ssc_circ_0437 from *ULK4,* two different primer sets for ssc_circ_1061 from *ZNHIT6* primer pair a and b*,* ssc_circ_1132 from *LIN7A,* two different primer sets for ssc_circ_1101 from *KHDRBS3*, primer pair a (which resulted in amplification of two splicing forms and was excluded) and b, and ssc_circ_0118 from *PDE10A* that resulted in amplification of two splicing forms (and excluded from further analysis).

Supplementary Figure S2. Figure showing validation of the set of circRNAs by Sanger sequencing. **A.** ssc_circ_1141 from *PTGES3.* **B**. ssc_circ_0670 from *BAZ2B.* **C.** ssc_circ_0437 from *ULK4.* **D.** ssc_circ_1061 from *ZNHIT6.* **E.** ssc_circ_1101 from *KHDRBS3*.

Supplementary Figure S3. Summary outline of the different steps of the analysis. Framework of the dataset and analyses included in the study.

Supplementary Figure S1

A

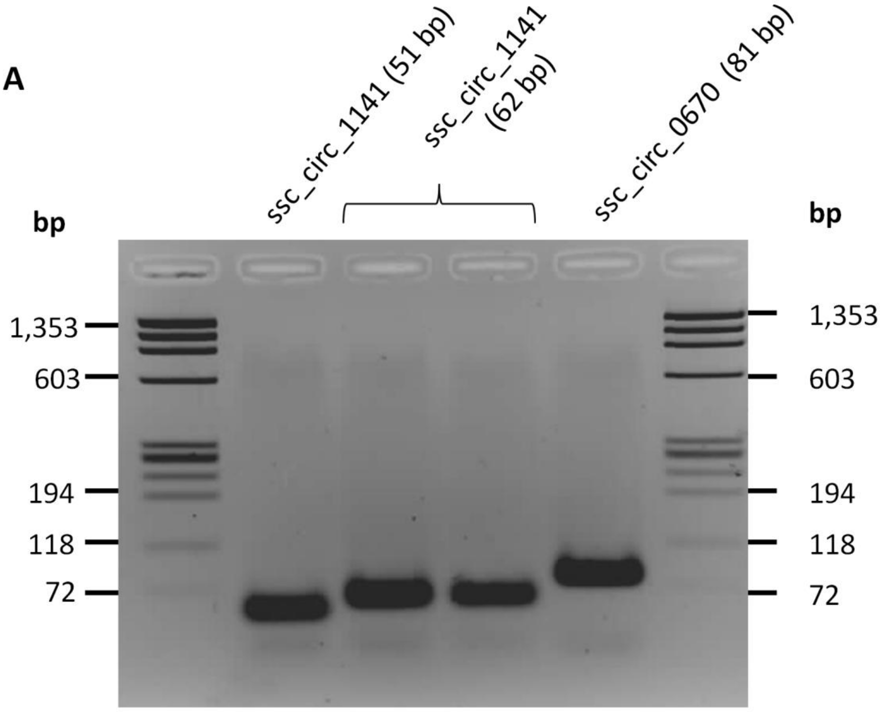

B

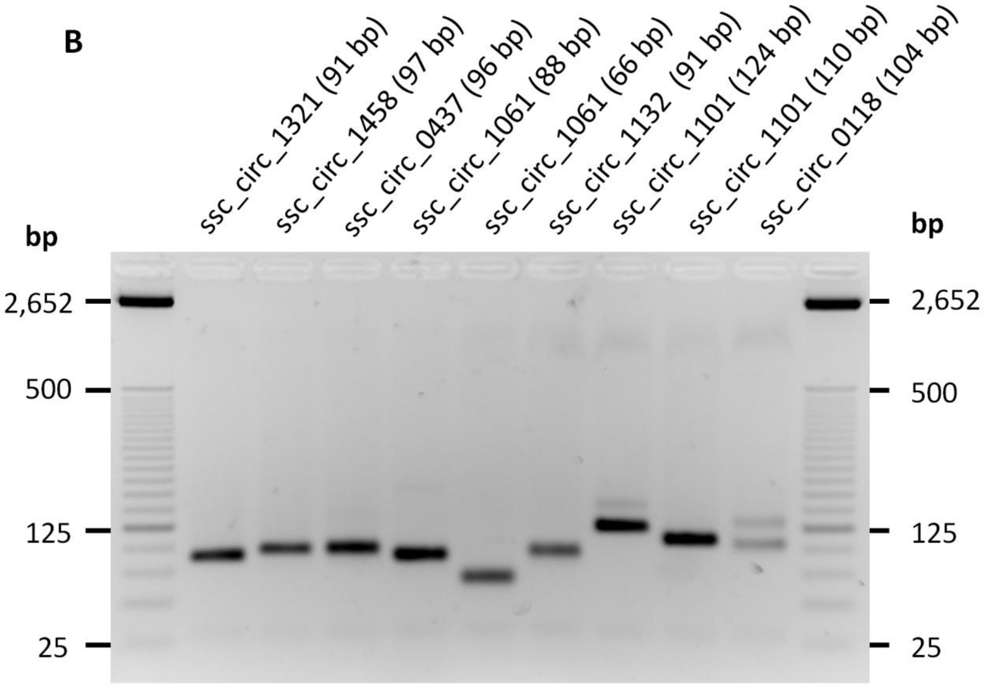

Supplementary Figure S2

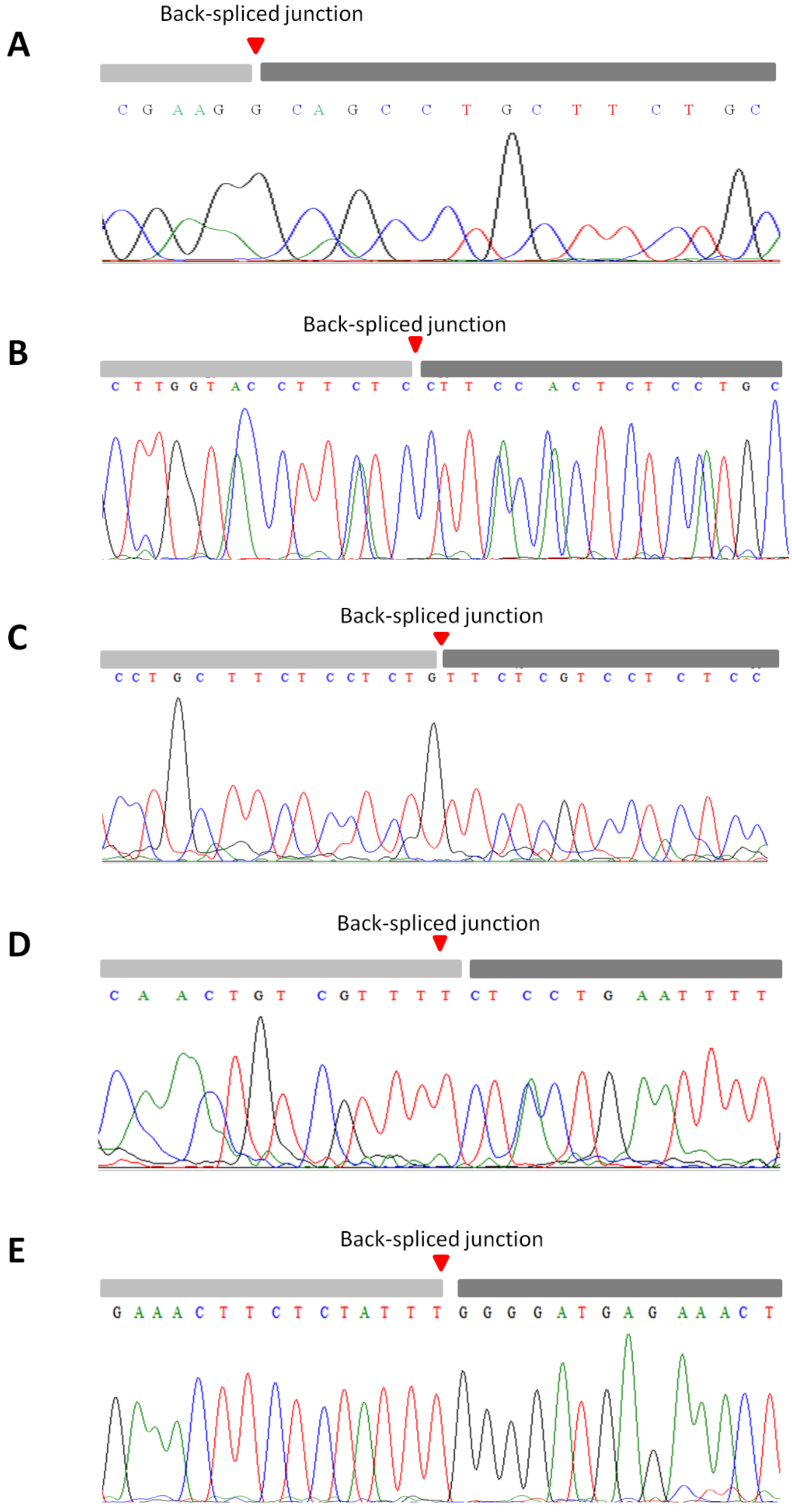

Supplementary Figure S3

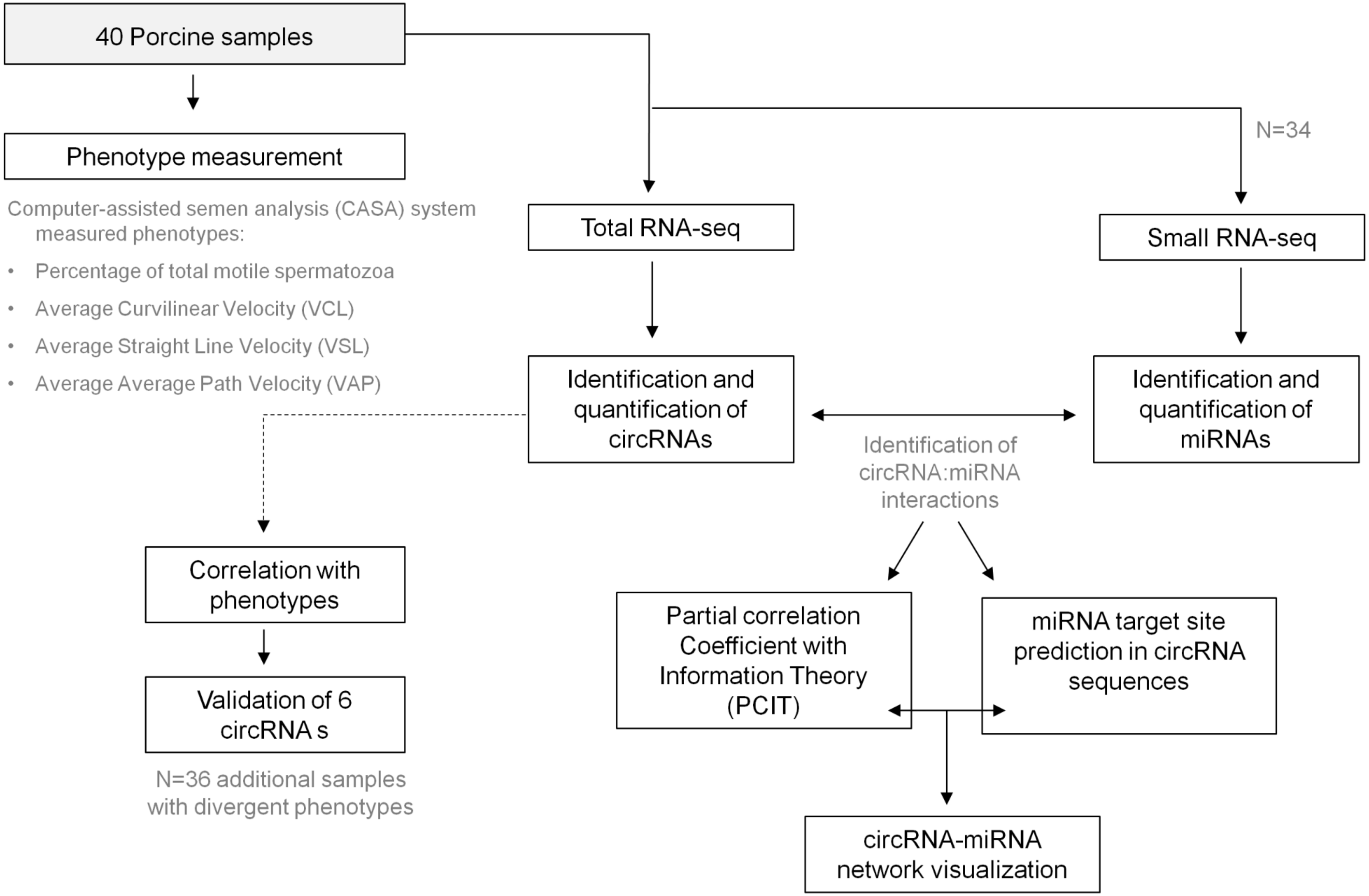

Supplement: Supplementary file 1 — Supplementary Information. [file 41598_2020_64711_MOESM1_ESM.pdf]
